# Consent for Use of Clinical Leftover Biosample: A Survey among Chinese Patients and the General Public

**DOI:** 10.1371/journal.pone.0036050

**Published:** 2012-04-27

**Authors:** Yi Ma, HuiLi Dai, LiMin Wang, LiJun Zhu, HanBing Zou, XianMing Kong

**Affiliations:** 1 Department of Biobank, Renji Hospital, Shanghai JiaoTong University School of Medicine, Shanghai, China; 2 Department of Science and Study, Renji Hospital, Shanghai JiaoTong University School of Medicine, Shanghai, China; University Hospitals of Geneva, Switzerland

## Abstract

**Background:**

Storage of leftover biosamples generates rich biobanks for future studies, saving time and money and limiting physical impact to sample donors.

**Objective:**

To investigate the attitudes of Chinese patients and the general public on providing consent for storage and use of leftover biosamples.

**Design, Setting and Participants:**

Cross-sectional surveys were conducted among randomly selected patients admitted to a Shanghai city hospital (n = 648) and members of the general public (n = 492) from May 2010 to July 2010.

**Main Outcome Measures:**

Face-to-face interviews collected respondents-report of their willingness to donate residual biosample, trust in medical institutions, motivation for donation, concerns of donated sample use, expectations for research results return, and so on.

**Results:**

The response rate was 83.0%. Of the respondents, 89.1% stated that they completely understood or understood most of questions. Willingness to donate residual sample was stated by 64.7%, of which 16.7% desired the option to withdraw their donations anytime afterwards. Only 42.3% of respondents stated they “trust" or “strongly trust" medical institutions, the attitude of trusting or strongly trusting medical institutions were significantly associated with willingness to donate in the general public group.(*p*<0.05) The overall assent rate for future research without specific consents was also low (12.1%). Hepatitis B virus carriers were significantly less willing than non-carriers to donate biosamples (32.1% *vs.* 64.7%, *p*<0.001).

**Conclusions:**

Low levels of public trust in medical institutions become serious obstacle for biosample donation and biobanking in China. Efforts to increase public understanding of human medical research and biosample usage and trust in the ethical purposes of biobanking are urgently needed. These efforts will be greatly advanced by the impending legislation on biobanking procedures and intent, and our results may help guide the structure of such law.

## Introduction

Human biological material, including tissues, blood and other body fluids and excretions, has emerged as an important tool for biomedical research [1], often supplementing and sometimes replacing the animal or cell culture based research models. The biobanks that store such samples are rapidly evolving into rich resources for new and on-going studies, providing biosamples that may be investigated by new next generation technologies or reassessed as validation cohorts in established studies. Moreover, biobanks are particularly useful for scientists and clinicians lacking direct access to human specimens or a large enough condition-specific donor population. However, the inception of the biobanking industry raised many ethical and legal concerns and much governmental effort worldwide has been focused on establishing appropriate laws and regulations. China, in particular, has yet to pass such legislation, and even the informed consent process for initial donation of biosamples is relatively new. Ultimately, the success of such legislation and the public's willingness to participate in biosample donation and biobanking will be affected by issues of informed consent type (broad or specific) [2]–[4], benefit sharing [5], [6], individual privacy protection [7], information access [8], [9] and public trust [9], [10].

The recent phenomenon of globalization has corresponded with a trend towards resource sharing. Biobanks are often sought out for international collaborations so that researchers may obtain more diverse and larger sample sets, thus generating more clinically applicable results [11]. Unfortunately, the current fragmentations of the ethical and legal regulatory systems concerning biobanks, especially in countries like China, present serious obstacles for international medical research efforts [1], [12]. Recently, however, it has been raised to establish broad guidelines on conducting ethical and appropriate international biobank research [12], [13]. China is the world's most populated country, and as such has the potential to generate the world's largest human biobank. The feasibility of such an endeavor remains unknown due to the paucity of data on the citizen's attitudes towards biosample donation and storage. If the majority is unwilling to donate or consent to biobanking, China may not only lose out on international collaborations but also hinder human health advances worldwide. Strong legislation for biobanking will not only serve to ensure international collaborators of the integrity of Chinese biosamples and the Chinese research efforts involving other nations' biosamples, but also provide justification for greater public trust in biobanking.

To address these issues, we conducted cross-sectional surveys of Chinese attitudes towards biosample donation and human medical research. The surveys were carried out among hospital patients and the general public. Qualitative data from face-to-face interviews was assessed to determine willingness to donate biosamples and consent to biobanking, and to identify the factors influencing compliance or resistance.

## Methods

### Ethics Statement

The study was approved by the ethics committee of Renji hospital, and the survey qualified as involving only “minimal risks" to participants. The survey was completely anonymous and questionnaire responses were not linked with the participants' identification in survey process, so we didn't obtained informed consent. However, a verbal informed consent regarding the goals of the study and the willingness to participate was given to the potential respondents. This procedure was approved by the ethics committee of Renji hospital.

### Participant recruitment and selection

Two groups were enrolled in the study: hospital patients with diverse medical conditions and individuals from the general public.

Patients were recruited between May 2010 and June 2010 at the Shanghai Renji hospital, a university-affiliated teaching general hospital in Shanghai's Pudong district with more than two-million outpatients attended annually. Patients were recruited from each of 16 outpatient departments and had traveled from 12 provinces to seek treatment. Patients were selected for recruitment by using a random number table. The only inclusion criterion for study enrollment was age ≥16 years. Any critically ill patient who was unable to make decisions independently was excluded from participation.

The general public group was recruited between June 2010 and July 2010 in two rural communities (Waigang in West Shanghai, and Yinhang in North Shanghai) and three urban communities (Tangqiao, Jinyang and Weifang in central Shanghai city). Participants were selected for recruitment by using multistage stratified probability sampling; communities were selected at the first stage, followed by household addresses selected at the second stage and recruitment of all individuals ≥16 years living at a single address. Stratification was based on geographic areas. The face-to-face interviews were mainly conducted on Saturday or Sunday in community health centers, in order to avoid over-representation of unemployed individuals. Any individuals unable to care for themselves and to make decisions independently were excluded from enrollment.

### The questionnaire and the survey process

The survey was composed of questions aimed to determine the respondents' willingness to donate residual biosamples to a biobank, trust in medical institutions, motivation for donation, concerns of donated sample use, expectations for research results return, and perceptions about future research consent and commercial research. The survey questions were designed according to relevant literature review and the results from a pre-survey. The pre-survey was composed of 11 open-ended queries and was conducted on 50 patients and 44 general public members. The subsequent formal survey included all the 11 queries, which were related to the following contents: 1. Willingness to donate (Likert scales). 2. Donation withdrawal (Likert scales). 3. Concerns about donation (open-ended). 4. Level of trust in medical institutions (Likert scales). 5. Most trusted institutions (open-ended). 6. Motivation for donation (open-ended). 7. Anonymous donation (Likert scales). 8. Individual results return (Likert scales). 9. Willingness to donate for profit-making research (Likert scales). 10. Consent for future research (Likert scales). 11. Understanding of the above questions (Likert scales). (See details in figure legends and table footnotes)

The survey process was facilitated by face to face interviews by using questionnaire in a small, private room. We totally had four interviewers and each interviewer was trained for five times to ensure they understood this survey enough. For each training session, we read 2–3 related paper carefully. Finally, the outline of the interview and every question of the questionnaire were discussed adequately. The interview outline is as follows: 1. Value of the biosample derived from human body. 2. Current situation of biosample donation. 3. Protection of donors' identities. 4. Explanation of the hypothetical scenario (see below). 5. Read and explain the questions in questionnaire. The respondents were asked to indicate their willingness to donate biosamples to an existing biobank (Renji biobank, established in 2008) based on the following hypothetical scenario: “The doctors at this hospital plan to carry out medical research on a type of cancer treatment. If you were a cancer patient, would you be willing to donate the leftover biosample from your diagnosis or surgical treatment for this research?"

All verbal portions of the survey were conducted in plain Chinese. For the close-ended questions (with multiple choice answers), respondents read the options, and then gave verbal answers that were recorded by the interviewers. If the respondents were illiterate, one of the interviewers would read aloud both the questions and answer options. Features of every question were discussed adequately to ensure understanding of a respondent's intent, and respondents were given the opportunity to ask questions freely. Interviews lasted between 10 and 15 minutes. At the end, every participant was asked to indicate their understanding of all the questions in the survey by using a 5-point Likert scale that ranged from “totally understood" to “totally did not understand".

### Statistical analysis

The associations of donation willingness, expectation for the research results to be returned to the individual, willingness to give future research consent, and current trust in medical institutions with demographic and other attitudinal factors were determined by comparing proportions and prevalence ratios using the Chi-square test. If a statistical significance of 0.05 or less was detected, it would be entered into a regression model. Multiple logistic regression was used to examine demographic and other attitudinal factors associated with people's willingness or their other preferences. This multivariable statistical analysis yields the ratios of willingness adjusted for all other confounding variables included in the regression analysis. Consent was coded as “1", while consent denial and undecided was coded “0"; thus, the odds ratios were interpreted as the odds of consent. The tests were two-tailed, and *p*<0.05 was considered significant. All data analyses were performed with the Statistical Package for the Social Sciences, version 16.0 (SPSS, Chicago, IL, USA). Data was plotted by Sigmaplot, version 11.0 (Systat Software, San Jose, CA, USA).

## Results

### Response rates and respondents characteristics

In total, 648 patients and 492 general public members (age range: 16–82 years) were enrolled. Of those, 531 (81.9%) patients and 415 (84.3%) general public members completed the questionnaire. The unanswered questions on this survey varied from 0 to 7 (mean, 2.1). The respondents' general characteristics are shown in Table S1.

### Willingness to donate

About 64.7% of respondents (67.0% of patients and 61.7% of general public members) were willing to donate their tissue for research, while 28.9% (25.2% and 33.5%, respectively) refused to donate and 6.4% (7.7% and 4.8%, respectively) were undecided. Of those willing to donate, 16.7% (16.9% and 16.4%, respectively) stated a desire to withdraw their donations afterwards. The patients were slightly more willing to donate than the general public members, but the difference was not statistically significant (67.0% *vs.* 61.6%, *p* = 0.08). The respondents from urban areas were, however, significantly more willing to donate than the rural respondents (71.65% *vs.* 45.96%, *p*<0.001), probably due to higher education levels for the urban people (college or higher education: 49.21% *vs.* 42.24%), although the difference was not significant (*p* = 0.16).

Willingness to donate residual sample differed significantly by age, employment, and educational level in both the patient and general public groups. Respondents who were younger, students, company employed, or had a higher education level were more willing to donate residual biosamples (Table S1). The willingness to donate a residual biosample was independently associated with an individual's trust in medical institutions, hepatitis B virus-negative status, or having no concerns about the donation (concern #9) (*p*<0.05) (Table 1).

**Table 1:** Results of multiple logistic regressions examining demographic and attitudinal differences in preferences to donate biosample.

			*p*	Odds ratio	95% CI
Patients	Age	Age	<0.001	0.96	0.94–0.98
	Stigmatizing health conditions	Hepatitis B virus carriers(yes/no)	<0.001	0.22	0.09–0.48
	Do you trust medical institutions	Strongly trust	0.81	-	-
		Trust	0.76	-	-
		General	RE	-	-
		Mistrust	0.04	0.40	0.17–0.94
		Strongly mistrust	0.003	0.19	0.07–0.56
	Concerns*	Concern #1(yes/no)	0.03	2.00	1.06–3.76
		Concern #9(yes/no)	<0.001	14.19	3.96–50.81
General public	Geographic areas	Rural areas	<0.001	0.38	0.22–0.66
		Urban areas	RE		
	Stigmatizing health conditions	Hepatitis B virus carriers(yes/no)	0.004	0.20	0.07–0.60
	Do you trust medical institutions	Strongly trust	0.07	-	-
		Trust	0.03	1.96	1.09–3.52
		General	RE	-	-
		Mistrust	0.88	-	-
		Strongly mistrust	0.96	-	-
	Concerns*	Concern #3(yes/no)	0.04	0.45	0.21–0.97
		Concern #5(yes/no)	0.04	0.40	0.17–0.97
		Concern #9(yes/no)	0.001	8.98	2.58–31.23

Demographic items were excluded from this table if none was statistically significant. Except age, all variables were entered into the models as categorical variables.

CI: Confidence Interval.

RE: Reference.

* : Concern 1–9: 1: more tissue would be taken for research than was needed. 2: my confidentiality would be lost. 3: donations might be used in research that is dangerous to me or others. 4: donation might spread my disease. 5: donation might cause potential ethical issues. 6: I do not trust the intent of medical institutions. 7: I haven't thought about donation. 8: it's bad for my health. 9: no concern.

### Consent for future research or profit-making research

**Figure 1:**
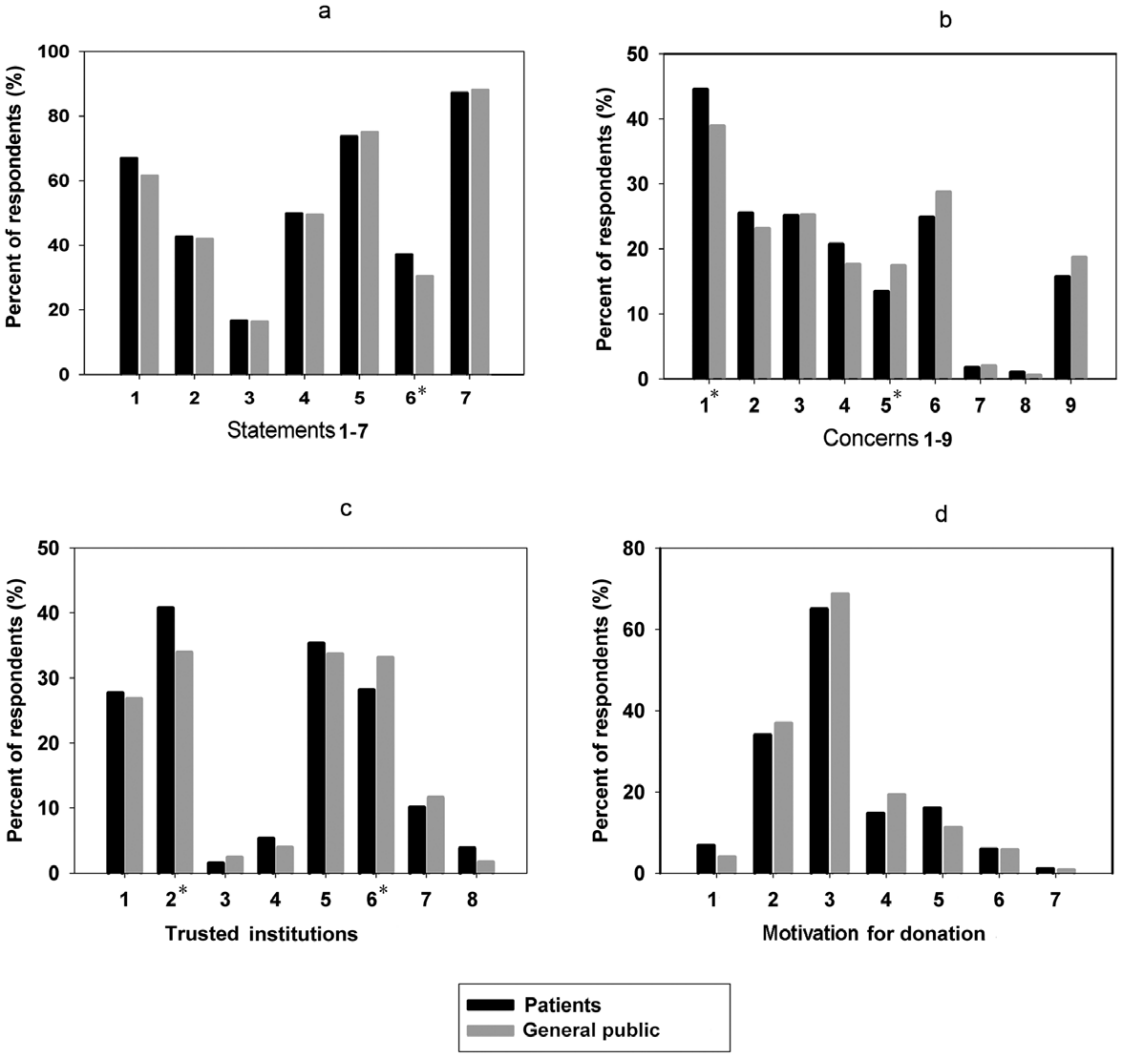
The results of χ^2^-tests examining the differences in attitudes and perceptions between patients and general public members. *: P<0.05. a. Statements 1–7: 1: I'm willing to donate the residual sample. 2: I trust medical institutions. 3: I would want to withdraw my donation afterwards. 4: I would want to donate anonymously. 5: I would want to know my individual result. 6: I would like my sample to be used for profit-making research. 7: I would not like my sample to be used for future research without my specific consent. b. Concerns about donation of biosample 1–9: 1: more tissue would be taken for research than was needed. 2: my confidentiality would be lost. 3: donations might be used in research that is dangerous to me or others. 4: donation might spread my disease. 5: donation might cause potential ethical issues. 6: I do not trust the intent of medical institutions. 7: I haven't thought about donation. 8: it's bad for my health. 9: no concern. c. Trusted institutions 1–8: 1: university research institutions. 2: hospital research institutions. 3: for-profit company research institutions. 4: ethics committee or IRB. 5: Chinese medical association. 6: government research institutions (such as, the health bureau). 7: no trusted institutions. 8: other institutions. d. Motivation for donation 1–7: 1: to establish a good relationship with medical institutions. 2: it's my obligation. 3: to benefit future patients. 4: to benefit me and my family. 5: no motivation. 6: can't think of a reason to refuse. 7: other motivation.

There was an overall low assent rate (12.1%) for future research without specific consent (Figure 1a). No demographic or attitudinal factor was independently associated with consent for future research (data not shown). As for attitudes about profit-making research, only 34.3% of all willing donors expressed agreement to their sample being used for profit-making research. Patients were more willing to donate for profit-making research (*p*<0.05) (Figure 1a).

### Expectation for research results to be returned to the individual

Most respondents (74.3%) wanted to have the research results obtained from their individual donated sample to be returned to them (Figure 1a). No significant differences were found between those wanting research results returned among the general public members (75.0%) or hospital patients (73.9%) (Table S1). Being company employed, however, was significantly associated with wanting research results returned (Odds ratio: 2.98, 95% CI: 1.26–7.02, *p* = 0.01).

### Concerns about biosample donation

The respondents indicated nine types of concerns, which are listed in Figure 1b. Patients were more concerned about “more tissue would be taken for research than was needed" (#1) (*p*<0.05), while general people were more concerned about “donation might cause potential ethical issues" (#5) (*p*<0.05) (Figure 1b). Lack of concern (#9) was found to be independently associated with willingness to donate in both the patient and general public groups. The concern types involving dangerous research (#3) and ethical issues (#5) were significantly associated with unwillingness to provide biosamples in the general public group (*p*<0.05) (Table 1).

### Trust in medical institutions

Only 42.3% of total respondents stated that they “trust" or “strongly trust" medical institutions to manage their donations properly (Figure 1a), while 43.9% of respondents stated “neutral" and 13.8% stated either “mistrust" or “strongly mistrust". Respondents who chose “trust" or “strongly trust" were significantly more likely to have no concern about the biosample donation, as compared to those who chose “mistrust" or “strongly mistrust" (21.5% *vs.* 0.8%, *p*<0.001). Only one respondent didn't trust medical institutions and chose the option “I have no concern about donation". Given the choice of which type of institutions were most trustworthy to manage biosamples, the majority of respondents chose hospital research institutions (37.7%), followed closely by Chinese medical association (34.6%), and government institutions (30.3%). Only 4.7% of the total respondents chose management by ethics committee or IRB, and even fewer (2.0%) chose the for-profit company research institutions (Figure 1c). Patients were more likely to trust hospital research institutions (*p*<0.05), while general people were more likely to trust government institutions (*p*<0.05) (Figure 1c).

After adjusting for potentially confounding factors, the attitude of trusting medical institutions were significantly associated with willingness to donate in the general public group (*p*<0.05) (Table 1).

### Motivation for donation

Most willing donors reported their motivation as being “to benefit future patients" (66.5%), with the next most common attitude being “it's my obligation" (34.6%). Fewer of the general public members agreed with the motivation “to establish a good relationship with medical institutions" than did the patients, but the difference was not statistically significant (6.74% *vs.* 3.90%, *p* = 0.13) (Figure 1d).

### Attitudes of respondents with stigmatizing health conditions

We surveyed four kinds of potentially stigmatizing health conditions: hypertension, diabetes mellitus, hepatitis B, and depression (Table S1). Respondents with the stigmatizing health conditions were less willing to donate their leftover tissue than those without (51.1% *vs.* 64.7%, *p*<0.001). However, when the respondents with hepatitis B were removed from the analysis of stigmatizing health conditions *vs.* unaffected individuals the significant difference was lost (59.8% *vs.* 64.7%, *p* = 0.20).

Hepatitis B is a chronic infectious disease with high incidence in China. One hundred and sixty-eight respondents (8.9%) in this survey were hepatitis B virus carriers, and this group was significantly less willing to donate biosamples than those with any other stigmatizing health conditions, among both the patient and general public groups (*p*<0.05) (Table S1).

### Participants' understanding of the survey questions

Of the total respondents, 45.1% stated they understood the questionnaire completely, while 45.2% stated they understood more than two-thirds of the questions. Only 7.0% stated that they were only able to understand about one-half of the questions, and 2.6% stated that they had understood less than one-third or none of the questions at all.

## Discussion

Although the ownership of donated biosamples remains a controversial subject, it is recognized that patients have the autonomy to determine the present and future use of their donations. Respect for autonomy is one of the fundamental guidelines in biosample donation management, and doctors should carefully interact with their patients to facilitate an informed choice being made without influencing the patient's decision. Patients' confidence can only be maintained and further developed if health care professionals attach more importance to their autonomy. Members of the general public are the potential biosample donors, respect for autonomy not only promote the public trust in biobanks and medical institutions, but also encourage them to donate biosamples. Since biobank legislation aims to ensure respect of donors' autonomy and protect them from potential harm (whether it be physical or perceived) [14], it is necessary to understand the donors' attitudes towards biosample donation and specific concerns.

This is the first study to examine attitudes of Chinese patients and general public members towards donating and biobanking biological samples. The overall results revealed a low willingness to donate, a low level of trust in medical institutions and ethics committees, and a low assent rate for future research without specific consent. The diversity of our study participants (patient/general public, urban/rural, ranges of existing medical conditions and age) corresponded with all the people who are potential biosample donors.

### Principal findings and differences with other studies

The level of willingness to donate found in our study (64.7%) was lower than that reported by other similar studies in the literature (70–90%) [15]–[19]. Furthermore, 16.7% of our respondents who were willing to donate said they would withdraw their donations at some point in the future; hence, the actual number of respondents who were willing to donate biosamples was less than one-half. In addition, people being surveyed were just put into the hypothetical position of cancer patients, which might make them more willing to donate, making the low rates even more striking.

The results showed that respondents with higher education levels were more willing to donate biosamples. The finding was similar to the study reported among the general population in USA [20]. Our survey also revealed that unwillingness to donate in the Chinese population was based on a general low level of trust of medical institutes and hepatitis B virus-positive status. Public trust has been previously identified as a significant influencing factor for the individual's decision to provide a biosample donation [5]; it is generally believed that securing public trust and confidence is a necessary step towards ensuring the long-term viability of biobanks [21]. Because most biobanks in China were established by hospitals in the past few years, and the survey on patients was conducted in the hospital and it was based on the hypothesis that the biosamples would be stored at an existed biobank hosted by the hospital. So we examined the respondents' trust in medical institutions (hospital) in this study and we found that only 42.3% of total respondents trusted medical institutions to manage their donations properly. Decreased confidence in medical institutions over time will likely have damaging consequences on biosample donation.

We would like to speculate on the causes of this loss in public trust of the medical institutions in China. Several possible reasons immediately come to mind. First, the unequal health care system. Health care has become the number-one cited concern of China's population in recent years [22]. However, the high out-of-pocket cost and limited insurance coverage [23] had caused a disparity in access to healthcare. In addition, the health workforce is maldistributed among the nation, physically limiting access to some (rural) populations and widening the gap in health status across the nation [24]. This situation has been compounded by Chinese popular media outlets reporting sensationalized stories of medical malpractice, causing the public's mistrust of doctors and hospitals [25]. In addition, many patients believe that doctors provide multiple tests in order to obtain additional benefits from their employers or insurers, while doctors have defensively argued that these tests are confirmatory and actually protect patients from misdiagnosis [25]. Unfortunately, the current trust between doctors and patients is eroded, and the physician-patient relationship is entering a vicious circle in China.

In the present study, respondents who trusted medical institutions were more willing to donate biosamples, which might be due to their lack of concern about the eventual use or outcome of the donation. The respondents' trust was found to be strongest in hospital research institutions, the Chinese medical association and government's institutions. A minimal amount of respondents (4.7%) preferentially trusted an ethics committee or IRB. It is possible that the respondents mistrust the management role for ethics committees, but it is also possible that they were most familiar with hospitals, and that most did not truly understand what an ethics committee or an IRB were. However, this finding may also reflect the ethics committee or IRBs not having contributed significant activities to the general public. China's ethics committee and IRBs should do more to increase their influence, especially for those not completely independent of their affiliated research institutes.

Previous studies have found that people with stigmatizing health conditions may have different opinions of sample donation [26]. In this study, Respondents with the stigmatizing health conditions were less willing to donate their leftover tissue than those without. However, when the respondents with hepatitis B were removed from the analysis, the significant difference was lost. In China, an estimated 120 million people are infected with hepatitis B virus [27], accounting for 10% of the total population. In recent years, many hepatitis B virus carriers have experienced discrimination in the workplace or in social situations, and this trend appears to be increasing. In this survey, 8.9% of the respondents were hepatitis B virus carriers. We found that hepatitis B virus carriers were more unwilling to donate biosamples than the rest of the respondents. This sub-population should then be a focus group of programs to promote confidence in the medical community and human medical research and of doctors in the efforts to establish more productive and respectful communications.

Overall, only 12.1% of the respondents preferred the option to authorize any future research on their individual biosamples. This percentage was lower than that reported from the other similar studies in the literature [3], [8], [28], [29]. The general low level of trust in medical institutions might be a reason why most participants want to control the future usage of their donations. Thus, specific informed consent might be a better method than broad consent to encourage Chinese to donate biosamples; not only will this approach protect patients' interests and limit potential access to personal information, but it will ensure donor autonomy. Furthermore, by gaining specific consent for every future study the public trust in biobanks and medical researchers will be consistently renewed.

The strengths of this study were the high response rate, the diverse population examined, and the integrity of the data gathered from face-to-face interviews; however, each of these features can also represent a weakness of the study. First, the non-response rate of 17% may have introduced some bias. Second, the patients were enrolled from only one general hospital in a specific geographic region, which may limit the generalizability of our results. Third, approximately 10% of the participants had difficulty understanding the questions even when fully explained during the face-to-face interview. In addition, participants' understanding of the questions was self-assessed which couldn't rule out misunderstandings. Finally, the survey on patients was conducted in hospital, face-to-face questionnaire might underestimate the reluctance to donate, and overestimate trust in medical institutions.

### Conclusions and policy implications

The willingness of Chinese patients and general public members to donate biosamples is low. Low level of trust in medical institutions has become an obstacle for initial donation and biobanking for future research. China should take immediate action to increase public trust in biobanking and willingness to donate. We believe that the findings from this study have important implications for China's biobanks legislation that is currently being constructed and for future international research collaborations.

## Supporting Information

Table S1 **The demographic/clinical details of respondents and their attitudes towards donation.**
(DOC)
