# Supplementary material for: Consent for Use of Clinical Leftover Biosample: A Survey among Chinese Patients and the General Public
Source: PLoS One. 2012 Apr 27;7(4):e36050. doi: 10.1371/journal.pone.0036050 (PMC3338618; doi:10.1371/journal.pone.0036050)
Supplement: Table S1 — The demographic/clinical details of respondents and their attitudes towards donation. (DOC) [file pone.0036050.s001.doc]

|  |  |  |  | I’m willing to donate the residual sample. | | I would want to get my individual result. | | I would not like my sample to be used for future research without my specific consent. | | I trust medical institutions will manage my donation properly. | |
| --- | --- | --- | --- | --- | --- | --- | --- | --- | --- | --- | --- |
|  |  | Variable | N (%) | Agree N (%) | *p* | Agree N (%) ‡ | *p* | Agree N (%) ‡ | *p* | Agree N (%) § | *p* |
| Patients | Age | 16-29 | 138(26.0) | 108(78.3)* |  | 85(78.7) |  | 95(88.0) |  | 57(41.3) |  |
|  |  | 30-44 | 184(34.7) | 125(67.9) |  | 92(73.6) |  | 108(86.4) |  | 81(44.0) |  |
|  |  | 45-59 | 134(25.2) | 82(61.2) |  | 56(68.3) |  | 72(87.8) |  | 55(41.0) |  |
|  |  | 60- | 75(14.1) | 41(54.7) | 0.002 | 30(73.2) | 0.45 | 36(87.8) | 0.98 | 33(44.0) | 0.93 |
| General public |  | 16-29 | 94(22.8) | 68(72.3) |  | 53(77.9) |  | 59(86.8) |  | 41(43.6) |  |
|  |  | 30-44 | 138(33.4) | 87(63.0) |  | 66(75.9) |  | 81(93.1) |  | 58(42.0) |  |
|  |  | 45-59 | 121(29.3) | 68(56.2) |  | 48(70.6) |  | 60(88.2) |  | 50(41.3) |  |
|  |  | 60- | 60(14.5) | 32(53.3) | 0.047 | 24(75.0) | 0.79 | 29(90.6) | 0.59 | 24(40.0) | 0.97 |
| Patients | Gender | Men | 242(45.6) | 167(69.0) | 0.39 | 117(70.1) | 0.12 | 147(88.0) | 0.72 | 102(42.1) | 0.86 |
|  |  | Women | 289(54.4) | 189(65.4) | 0.39 | 146(77.2) | 0.12 | 164(86.8) | 0.72 | 124(42.9) | 0.86 |
| General public |  | Men | 249(60.0) | 151(60.6) |  | 114(75.5) |  | 129(85.4) |  | 106(42.6) |  |
|  |  | Women | 166(40.0) | 105(63.3) | 0.59 | 78(74.3) | 0.83 | 96(91.4) | 0.15 | 68(41.0) | 0.75 |
| Patients | Employment | Enterprise/company employed | 220(41.4) | 159(72.3) |  | 125(78.6) |  | 141(88.7) |  | 90(40.9) |  |
|  |  | Self-employed | 71(13.4) | 51(71.8) |  | 38(74.5) |  | 45(88.2) |  | 29(40.8) |  |
|  |  | Farmers | 54(10.2) | 25(46.3) |  | 16(64.0) |  | 22(88.0) |  | 19(35.2) |  |
|  |  | Students | 37(7.0) | 26(70.3) |  | 19(73.1) |  | 21(80.8) |  | 18(48.6) |  |
|  |  | Retired | 93(17.5) | 56(60.2) |  | 40(71.4) |  | 48(85.7) |  | 46(49.5) |  |
|  |  | Unemployed | 56(10.5) | 39(69.6) | 0.006 | 25(64.1) | 0.39 | 34(87.2) | 0.92 † | 24(42.9) | 0.56 |
| General public |  | Enterprise/company employed | 161(38.8) | 104(64.6) |  | 80(76.9) |  | 93(89.4) |  | 74(46.0) |  |
|  |  | Self-employed | 92(22.2) | 63(68.5) |  | 52(82.5) |  | 55(87.3) |  | 33(35.9) |  |
|  |  | Farmers | 32(7.7) | 16(50.0) |  | 12(75.0) |  | 15(93.8) |  | 12(37.5) |  |
|  |  | Students | 19(4.6) | 17(89.5) |  | 12(70.6) |  | 15(88.2) |  | 10(52.6) |  |
|  |  | Retirement | 78(18.8) | 37(47.4) |  | 24(64.9) |  | 31(83.8) |  | 30(38.5) |  |
|  |  | Unemployed | 33(8.0) | 19(57.6) | 0.004 | 12(63.2) | 0.33 † | 16(84.2) | 0.90 † | 15(45.5) | 0.53 |
| Patients | Education | Primary school or lower | 134(25.2) | 73(54.5)* |  | 54(74.0) |  | 60(82.2) |  | 56(41.8) |  |
|  |  | Secondary school | 139(26.2) | 95(68.3) |  | 68(71.6) |  | 84(88.4) |  | 69(49.6) |  |
|  |  | Junior college or higher | 258(48.6) | 188(72.9) | 0.001 | 141(75.0) | 0.83 | 168(89.4) | 0.28 | 101(39.1) | 0.13 |
| General public |  | Primary school or lower | 116(28.0) | 61(52.6) |  | 40(65.6) |  | 51(83.6) |  | 47(40.5) |  |
|  |  | Secondary school | 106(25.5) | 65(61.3) |  | 49(75.4) |  | 59(90.8) |  | 36(34.0) |  |
|  |  | Junior college or higher | 193(46.5) | 130(67.4) | 0.04 | 103(79.2) | 0.13 | 115(88.5) | 0.45 | 91(47.2) | 0.08 |
| Patients | Geographic areas | - | - | - | - | - | - | - | - | - | - |
| General public |  | Rural areas | 161(38.8) | 74(46.0)* |  | 74(46.0)* |  | 82(50.9)* |  | 64(39.8) |  |
|  |  | Urban areas | 254(61.2) | 182(71.6) | <0.001 | 143(56.3) | 0.04 | 172(67.7) | 0.001 | 110(43.3) | 0.47 |
| Patients | Duration of current disease | First visit | 147(27.7) | 100(68.0) |  | 72(72.0) |  | 93(93.0) |  | 57(38.8) |  |
|  |  | ＜6 months | 126(23.7) | 80(63.5) |  | 61(76.3) |  | 71(88.8) |  | 54(42.9) |  |
|  |  | ≥6 months and ≤2 years | 131(24.7) | 97(74.0) |  | 69(71.1) |  | 81(83.5) |  | 61(46.6) |  |
|  |  | ≥2 years | 127(23.9) | 79(62.2) | 0.17 | 62(78.5) | 0.65 | 66(83.5) | 0.15 | 54(42.5) | 0.63 |
| General public |  | - | - | - | - | - | - | - | - | - | - |
| Patients | Personal history of hospitalization | Yes | 220(41.5) | 145(65.9) |  | 105(72.4) |  | 124(85.5) |  | 94(42.7) |  |
|  |  | No | 310(58.5) | 210(67.8) | 0.66 | 158(75.2) | 0.99 | 186(88.6) | 0.40 | 131(42.3) | 0.91 |
| General public |  | Yes | 107(26.0) | 64(59. 8) |  | 45(70.3) |  | 55(85.9) |  | 38(35.5) |  |
|  |  | No | 304(73.8) | 191(62.8) | 0.58 | 145(75.9) | 0.37 | 168(88.0) | 0.67 | 132(43.4) | 0.15 |
| Patients | Family history of genetic disease | Yes | 70(13.2) | 47(67.1) |  | 32(68.1) |  | 43(91.5) |  | 29(41.4) |  |
|  |  | No | 461(86.8) | 309(67.0) | 0.99 | 231(74.8) | 0.33 | 268(86.7) | 0.36 | 197(42.7) | 0.84 |
| General public |  | Yes | 51(12.4) | 32(62.7) |  | 23(71.9) |  | 29(90.6) |  | 21(41.2) |  |
|  |  | No | 359(87.6) | 222(61.8) | 0.90 | 167(75.2) | 0.68 | 194(87.4) | 0.78† | 148(41.2) | 0.99 |
| Patients | Stigmatizing health conditions | Hypertension | 82(15.4) | 54(65.9) |  | 37(68.5) |  | 48(88.9) |  | 37(45.1) |  |
|  |  | Diabetes mellitus | 36(6.8) | 21(58.3) |  | 15(71.4) |  | 17(81.0) |  | 13(36.1) |  |
|  |  | Hepatitis B virus carriers | 46(8.7) | 13(28.3)* |  | 10(76.9) |  | 11(84.6) |  | 16(34.8) |  |
|  |  | Depression | 19(3.6) | 11(57.9) | 0.001 | 8(72.7) | 0.96 † | 9(81.8) | 0.82† | 9(47.4) | 0.58 |
| General public |  | Hypertension | 67(16.1) | 37(55.2) |  | 24(64.9) |  | 29(78.4) |  | 25(37.3) |  |
|  |  | Diabetes mellitus | 18(4.3) | 10(55.6) |  | 6(60.0) |  | 8(80.0) |  | 7(38.9) |  |
|  |  | Hepatitis B virus carriers | 38(9.2) | 14(36.8)* |  | 10(71.4) |  | 11(78.6) |  | 16(42.1) |  |
|  |  | Depression | 15(3.6) | 11(73.3) | 0.09 | 8(72.7) | 0.90 † | 10(90.9) | 0.87 † | 6(40.0) | 0.99 |
|  |  |  |  |  |  |  |  |  |  |  |  |

**Table S1.** **The demographic/clinical details of respondents and their attitudes towards donation.**

*: This demographic category differs significantly from other categories in the group (*p* < 0.05).

†: Fisher's exact test.

‡: Including “strongly agree” and “agree”.

§: Including “strongly trust” and “trust”.
